# Molecular transmission network analysis of HIV-1 among older adults in Central China: a cross-sectional study in Shaodong City

**DOI:** 10.3389/fmicb.2026.1876738

**Published:** 2026-08-19

**Authors:** Xiaobai Zou, Wei Liu, Jieyu Zhou, Jinghang Zhang, Jie Chen, Jingxin Li, Wenxiu Deng, Huan Chen

**Affiliations:** 1 Hunan Provincial Center for Disease Control and Prevention, Changsha, Hunan, China; 2 Shaoyang Center for Disease Control and Prevention, Shaodong, Hunan, China; 3 Shaodong Center for Disease Control and Prevention, Shaoyang, Hunan, China

**Keywords:** CRF85_BC, heterosexual commercial sex, molecular transmission network, older adults (60 years and above), transmitted drug resistance

## Abstract

**Objective:**

The ageing population poses emerging challenges to HIV prevention, particularly in regions with high proportions of older adults. We conducted a cross-sectional study of 513 newly diagnosed HIV-infected individuals in Shaodong City, Hunan Province, China, between 2021 and 2025, with a focus on individuals aged ≥60 years.

**Methods:**

HIV-1 pol sequences were amplified, sequenced, and analyzed for subtype distribution and transmitted drug resistance (TDR). Molecular transmission networks were constructed using a 0.5% genetic distance threshold, and clusters were classified as large (≥10 nodes), medium (5–9 nodes), or small (<5 nodes).

**Results:**

CRF85_BC was the predominant subtype (32.55%), and a total of 37 transmission clusters were identified, with the two largest clusters comprising 99 and 19 nodes, respectively. High-risk nodes, defined as those with a transmission network score (TNS) ≥ 0.75 and degree ≥4, were primarily older male farmers engaged in heterosexual commercial sex. The overall TDR prevalence was 10.53%, with NNRTI resistance predominating, and K103N being the most frequent mutation.

**Conclusion:**

Our findings indicate that HIV transmission among older adults exhibits substantial clustering, largely driven by commercial sexual activity, and highlight the importance of molecular network analysis for identifying high-risk individuals and guiding targeted prevention interventions. Continuous surveillance of TDR within active clusters is critical for optimizing antiretroviral therapy strategies and mitigating onward transmission.

## 1 Introduction

Human immunodeficiency virus (HIV) remains a major global public health threat. According to estimates from the World Health Organization (WHO), approximately 3,900,000 people worldwide were living with HIV by the end of 2022 (UNAIDS, 2023). Since the first reported case of HIV infection in China in 1985, the cumulative number of reported HIV cases in China had reached 1,200,000 by the end of 2022 (He and Detels, 2005; Lyu and Chen, 2019).

The global HIV epidemic is increasingly challenged by population ageing. In 2022, people aged 50 years and older accounted for 36% of China’s total population (China NBoSo, 2023). During the same period, the proportion of HIV-positive individuals in this age group increased from 22% in 2011 to 48% in 2022 (Yue et al., 2021). HIV prevention and control among older adults face multiple challenges. On the one hand, older adults often have limited awareness of HIV and low rates of condom use. On the other hand, underlying comorbidities and increased vulnerability to injury may increase their susceptibility to HIV infection (Han, 2023; Wang et al., 2020). In addition, HIV diagnosis is often delayed in this population, and the increasing involvement of older adults in commercial sexual activities may prolong transmission chains and enhance transmission efficiency.

The HIV epidemic in China is characterized by substantial regional heterogeneity in its distribution, transmission patterns, and epidemiological characteristics among high-risk populations (Rich et al., 2023). Shaodong, located in central Hunan Province, has a population of approximately 1.3 million, with people aged 50 years and older accounting for more than 35% of the local population, indicating a pronounced ageing trend. According to de-identified aggregate surveillance data from the HIV/AIDS Comprehensive Response Information Management System (CRIMS) of the China Disease Prevention and Control Information System, more than 800 people were living with HIV in Shaodong City during the study period, with an average annual increase of more than 8% over the past decade. Individuals aged 50 years and older accounted for more than 60% of reported HIV-positive cases, indicating a pronounced ageing pattern in the local HIV epidemic.

Traditional epidemiological studies often rely on self-reported information to infer HIV transmission routes, which may be affected by recall and reporting biases and thereby limit the implementation of precise interventions (Hassan et al., 2017; Dennis et al., 2021; Oster et al., 2018). Molecular transmission network analysis, based on viral genetic sequence data, provides a complementary approach for identifying potential transmission links and high-risk clusters. This approach has been increasingly used to monitor HIV transmission and drug-resistant spread and to inform targeted public health interventions (Ragonnet-Cronin et al., 2019; Fan et al., 2026; Wang et al., 2025; Li L. et al., 2025; Liu et al., 2025a; Zhang et al., 2026). Molecular transmission network analysis not only facilitates the identification of active transmission clusters but also provides a framework for monitoring the dissemination of transmitted drug resistance (TDR) within high-risk networks (Huang et al., 2025; Lin et al., 2025; Ma et al., 2020). Integrating TDR surveillance with molecular epidemiological investigations may improve the effectiveness of both prevention and treatment strategies.

Given the limited evidence regarding HIV infection and drug resistance among older adults, we investigate the molecular transmission networks of HIV-1 among older adults in Shaodong. This study aimed to characterize the HIV epidemic in this population, identify transmission patterns and high-risk groups, and provide empirical evidence for the development of targeted intervention strategies.

## 2 Method

### 2.1 Ethics

The research protocol, which was approved by the relevant institutional review boards or independent ethics committees, was conducted in accordance with standards for the protection of patient safety and welfare. It was also conducted in compliance with Good Clinical Practices and the principles of the Declaration of Helsinki and its amendments. The patients’ verbal consent for treatment was obtained by the attending medical professionals at the clinical sites, who were responsible for their care. The patients’ names were documented on a list with the doctors’ signatures, attesting to their consent for treatment. The study and the verbal consent procedure were approved by the Ethical Committee of Hunan Provincial Center for Disease Control and Prevention (ethical approval number: Hunan CDC IRB-2024-007-01). Informed consent was obtained from all participants.

### 2.2 Study participants and data collection

The present study population comprised all newly diagnosed HIV-infected individuals in Shaodong City between 2021 and September 2025 who had no prior history of antiretroviral therapy (ART). Venous blood was collected by the researchers, then plasma was isolated and stored at −80 °C. Concurrently, anonymous epidemiological information was gathered, including age, sex, place of residence, marital status, mode of transmission, and pre-treatment CD4 cell count. Demographic characteristics and transmission categories were obtained from routine epidemiological investigations. Local epidemiological background data, including the cumulative number of reported HIV cases, annual growth trend, and age distribution of reported cases in Shaodong City, were obtained from de-identified aggregate routine surveillance data in the CRIMS of China Disease Prevention and Control Information System.

### 2.3 HIV-1 RNA extraction, amplification, and sequencing

HIV-1 RNA was extracted from 200 μL of plasma samples by employing the RNA Extraction Kit (Ex-DNA/RNA Virus 4.0, Tianlong, Xian, China). As previously described, reverse transcription and nested PCR amplification were performed on the HIV-1 pol gene fragment (HXB2: 2253–3,262) (Fan et al., 2025). The analysis of PCR products was conducted by means of 1% agarose gel electrophoresis, and the purification and sequencing of amplified positive products were undertaken by Guangzhou Hailite Biotechnology Co., Ltd. (Catalog No.: SUPI-1010). The organisation of sequence databases involved the exclusion of duplicate sequences and those that failed quality control. In summary, the following criteria were applied for the exclusion of sequences: insufficient length, the presence of stop codons, adverse insertions/deletions, and hypermutations. Furthermore, codon positions associated with drug-resistant mutations were excluded based on the HIV drug resistance database^1^ in order to minimise potential confounding effects of convergent evolution on sequence analysis (Xu et al., 2024). All sequences that failed these quality control filters were removed, yielding the final set of sequences used for downstream analyses.

### 2.4 HIV-1 subtype identification

For subtype identification, only the quality-controlled sequences that were ≥1,000 bp in length and contained ≤5% ambiguous bases were included. No additional sequences were excluded based on these length and ambiguity criteria beyond those already removed in the previous quality control step. The reference of the HIV-1 pol gene fragment (HXB2: 2253–3,262) (Fan et al., 2025) was retrieved from the LANL HIV database.^2^ Multiple sequence alignment was performed with MAFFT v7.490 (Katoh and Standley, 2013) using the --auto option. Subtype classification was conducted using the HIV Sequence Subtyping module of the National Microbiology Data Center platform (NMDC).^3^ All initial subtype assignments were subsequently validated by maximum-likelihood phylogenetic tree construction with standard reference sequences from the LANL HIV Database.

### 2.5 HIV molecular transmission network analysis

HIV Trace (based on the Tamura-Nei93 model), was employed to calculate the genetic distances (GD) between the samples. A molecular transmission network was then constructed, with a genetic distance threshold of 0.5% being utilized (Kosa kovsky Pond et al., 2018). The HIV molecular transmission network was then visualized using the HIV Molecular Transmission Network module of the National Microbiology Data Center platform (NMDC) (see text footnote 3). According to the “Technical Guidelines for HIV Transmission Network Monitoring and Intervention” (2025 revised edition) published by the Chinese Center for Disease Control and Prevention, the network is comprised of a set of nodes, each representing one HIV sequence or one case, and an edge between two nodes indicating a transmission association between them. The degree of a node is defined as the number of edges connecting that node to other nodes. A higher degree indicates a closer association with others and a higher transmission risk. In this study, large transmission clusters were defined as clusters containing more than 10 nodes. The sequences were then categorized into large clusters (LCs, ≥10 nodes), medium clusters (MCs, 5–9 nodes), and small clusters (SCs, <5 nodes) based on node aggregation. Furthermore, individuals with a minimum of four links were identified as high-risk transmission individuals (Yuan F. S. et al., 2021).

In accordance with the criteria for identifying high-risk nodes as outlined in the 2025 Edition of the Guidelines for HIV Transmission, Network Surveillance and Intervention (China Centers for Disease Control and Prevention, 2025), in combination with the core indicators of this study—TNS (Transmission Network Score) and degree – high-risk individuals requiring priority attention were identified. TNS is indicative of a node’s relative ranking of transmission risk within the network; that is to say, higher scores indicate greater transmission potential. In this study, nodes with TNS ≥ 0.75 were classified as high-risk individuals within the molecular network. The degree of a node is defined as the number of connections it has with other potentially linked transmission individuals. Nodes with a degree of at least 4 were identified as core hubs within the transmission network, posing significant transmission risks.

### 2.6 Drug resistance analysis

Quality-controlled sequences were submitted to the Stanford University HIV Drug Resistance Database (see text footnote 1) for drug resistance analysis to identify resistance-associated mutations and to evaluate resistance to protease inhibitors (PIs), nucleoside reverse transcriptase inhibitors (NRTIs), and non-nucleoside reverse transcriptase inhibitors (NNRTIs). Based on the database scoring system, drug resistance was classified into five levels: susceptible (score <10), potential low-level resistance (score 10–14), low-level resistance (score 15–29), intermediate resistance (score 30–59), and high-level resistance (score ≥60). In this study, low-level resistance or higher was considered resistant.

### 2.7 Statistical analysis

Statistical analyses were performed using SPSS (version 18.0, IBM Corp., Armonk, NY, USA). Continuous variables with non-normal distributions were expressed as median (interquartile range, IQR), while categorical variables were presented as frequencies and percentages. Between-group differences were assessed using the chi-square test. Univariate and multivariate logistic regression models were employed to identify factors associated with clustering; variables with a *p* < 0.10 in the univariate analysis were subsequently entered into the multivariate model. A two-tailed *p* < 0.05 was considered statistically significant. Graphical visualizations were partially generated using R software (version 4.3.2), and figure compositions were arranged with Adobe Illustrator.

## 3 Result

### 3.1 Demographic characteristics

In this study, HIV-1 pol sequences were successfully obtained from blood samples of 513 HIV-infected individuals. These 513 cases represented a large majority of the total newly diagnosed HIV infections in Shaodong City during the study period (2021–2025), indicating that the sample is broadly representative of the local epidemic. Among them, 85, 111, 112, 121, and 84 cases were diagnosed with HIV infection in 2021, 2022, 2023, 2024, and 2025, respectively. Most of the subjects were male (75.83%, 389/513). The median age was 61 years (IQR: 53–69), and the age distribution was predominantly composed of individuals aged 60 years and older. Nearly half of the study population was married (46.78%, 240/513). Illiteracy and primary education levels constitute the highest proportion at 58.28% (299/513). Transmission through heterosexual contact accounted for 92.98% (477/513) of infections, while transmission through Men who have sex with men (MSM) accounted for 7.02% (36/513). The median CD4 was 318 cells/ml (IQR:204–515). The HIV subtypes were CRF85_BC, CRF08_BC, CRF07_BC, CRF01_AE, and other subtypes, accounting for 32.55% (167/513), 21.64% (111/513), 19.69% (101/513), 16.57% (85/513), and 9.55% (49/513), respectively (Table 1.).

**Table 1:** Factor associated with clustering of newly diagnosed HIV-1 patients in Shaodong between 2021 and 2025.

Categories	In cluster (N/%)	Total N/(%)	OR(95%CI)	
			Unadjusted	Adjusted
Sex				
Male	190 (75.70)	389 (75.83)	Ref	Ref
Female	61 (24.30)	124 (24.17)	1.01 (0.68–1.52)	0.88(0.47–1.64)
Age(years)				
≤44	17(6.77)	73(14.23)	0.23(0.13–0.42)^***^	0.62(0.26–1.48)
45–59	74 (29.48)	157 (30.60)	0.69 (0.46–1.01)	1.31(0.80–2.15)
≥60	160 (63.75)	283 (55.17)	Ref	Ref
Married status				
Single	17 (6.77)	61 (11.89)	Ref	Ref
Married	122 (48.61)	240 (46.78)	2.68 (1.45–4.95)^**^	1.18(0.50–2.77)
Divorced/widowed	112 (44.62)	212 (41.33)	2.90 (1.56–5.40)^**^	1.32(0.55–3.16)
Education				
Primary school/illiterate	162 (64.54)	299 (58.28)	Ref	Ref
Junior middle school	64 (25.50)	137 (26.71)	0.74 (0.49–1.11)	1.08(0.65–1.78)
High or technical secondary school	19 (7.57)	55 (10.72)	0.45 (0.25–0.81)^**^	0.77(0.35–1.66)
Junior college or above	6 (2.39)	22 (4.29)	0.32 (0.12–0.83)^*^	2.09(0.46–9.58)
Occupation				
Farmer	212 (84.46)	426 (83.04)	Ref	Ref
Household chores and unemployment	17 (6.77)	36 (7.02)	0.90 (0.46–1.79)	1.38(0.59–3.22)
Business Services	7 (2.79)	14 (2.73)	1.01 (0.35–2.93)	1.37(0.36–5.16)
Retired personnel	8 (3.19)	14 (2.73)	1.35 (0.46–3.95)	2.12(0.56–8.00)
Other	7 (2.79)	23 (4.48)	0.44 (0.18–1.10)	0.93(0.29–2.98)
Sexual contact				
Heterosexual non-commercial sex	48 (19.12)	116 (22.61)	Ref	Ref
Heterosexual commercial sex	167 (66.53)	308 (60.04)	1.68 (1.09–2.59)^*^	1.35(0.78–2.36)
Spouse/regular partner tested positive	32 (12.75)	53 (10.33)	2.16 (1.11–4.19)^*^	2.55(1.13–5.79)^*^
MSM	4 (1.59)	36 (7.02)	0.18 (0.06–0.53)^**^	0.31(0.08–1.21)
Drug resistance				
No	226 (90.04)	455 (88.69)	Ref	Ref
Yes	25 (9.96)	58 (11.31)	0.77 (0.44–1.33)	0.40(0.21–0.79)^**^
Subtype				
CRF85_BC	135 (53.78)	167 (32.55)	Ref	Ref
CRF08_BC	44 (17.53)	111 (21.64)	0.16 (0.09–0.27)^***^	0.13(0.07–0.24)^***^
CRF07_BC	33 (13.15)	101 (19.69)	0.12 (0.07–0.20)^***^	0.13(0.07–0.25)^***^
CRF01_AE	31 (12.35)	85 (16.57)	0.14 (0.08–0.24)^***^	0.13(0.07–0.26)^***^
Other	8(3.19)	49(9.55)	0.05(0.02–0.11)^***^	0.04(0.02–0.11)^***^
CD4(cells/ml)				
<200	49 (19.52)	123 (23.98)	0.62 (0.38–1.02)	0.54(0.30–0.99)^*^
200~	82 (32.67)	157 (30.60)	1.03 (0.65–1.63)	1.04(0.59–1.82)
350~	50 (19.92)	97 (18.91)	1.00 (0.60–1.69)	0.78(0.42–1.44)
≥500	70(27.89)	136(26.51)	Ref	Ref

*p < 0.05, **p < 0.01, ***p < 0.001.

### 3.2 Multi-factorial analysis of clustering influence factors

This study employed a 0.5% genetic distance threshold to construct a molecular network, with a total of 251 subjects enrolled, yielding an enrolment rate of 48.93% (251/513). The enrolled cohort was predominantly comprised of males, individuals aged 60 years or older, those infected through non-marital commercial heterosexual intercourse, farmers, married individuals, and those with the CRF85_BC sub-type. Multivariate logistic regression analysis revealed higher recruitment rates among individuals with Spouse/regular partner who tested positive (OR:2.55, 95% CI: 1.13–5.79) than in those infected through heterosexual non-commercial sex, individuals with HIV drug resistance (OR:0.40, 95%CI:0.21–0.79) had significantly lower odds of clustering than those with HIV drug susceptibility. The enrollment rate was found to be lower in subjects with CRF08_BC (OR:0.13, 95%CI:0.07–0.24), CRF07_BC (OR:0.13, 95%CI:0.07–0.25), CRF01_AE (OR:0.13, 95%CI:0.07–0.26) and other subtypes (OR:0.04, 95%CI:0.02–0.11) than in those with CRF85_BC. Patients with CD4 < 200 cells/ml (OR:0.54, 95% CI: 0.30–0.99) had a significantly lower enrollment rate than those with CD4 ≥ 500 cells/ml. Please refer to Table 1 for further information.

### 3.3 Characteristics of LCs and MCs

**Figure 1:**
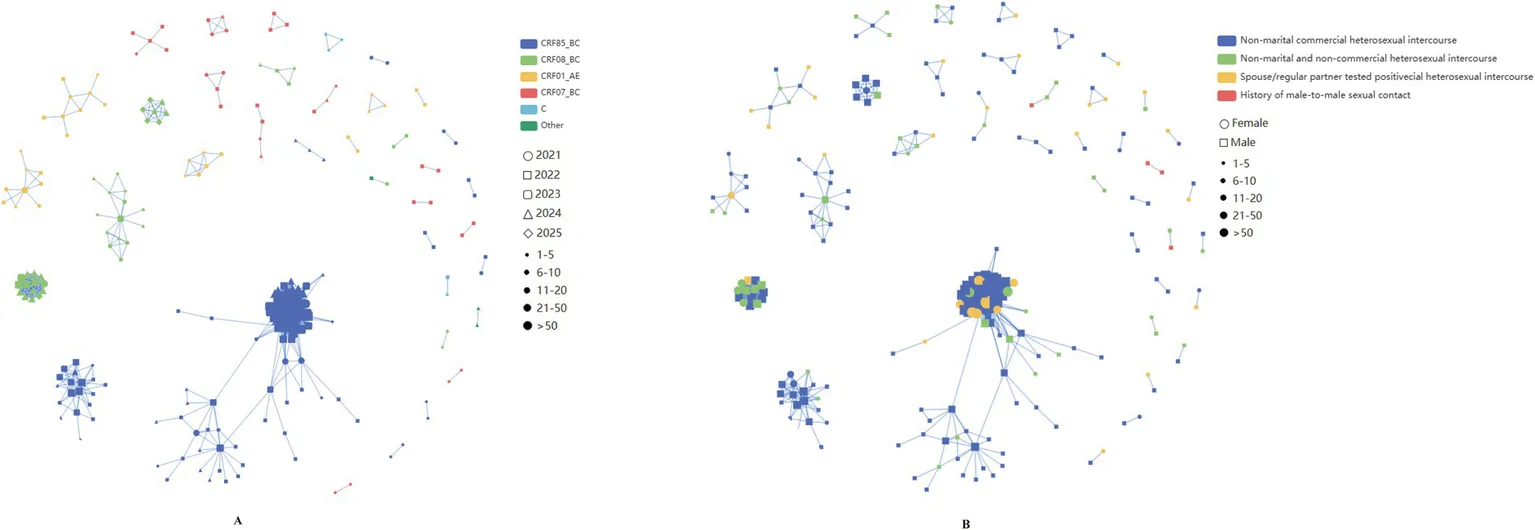
Molecular transmission network among newly diagnosed HIV-1 infections in Shaodong. **(A)** Transmission network by subtype and diagnosis year. **(B)** Transmission network by gender and sexual contact.

**Figure 2:**
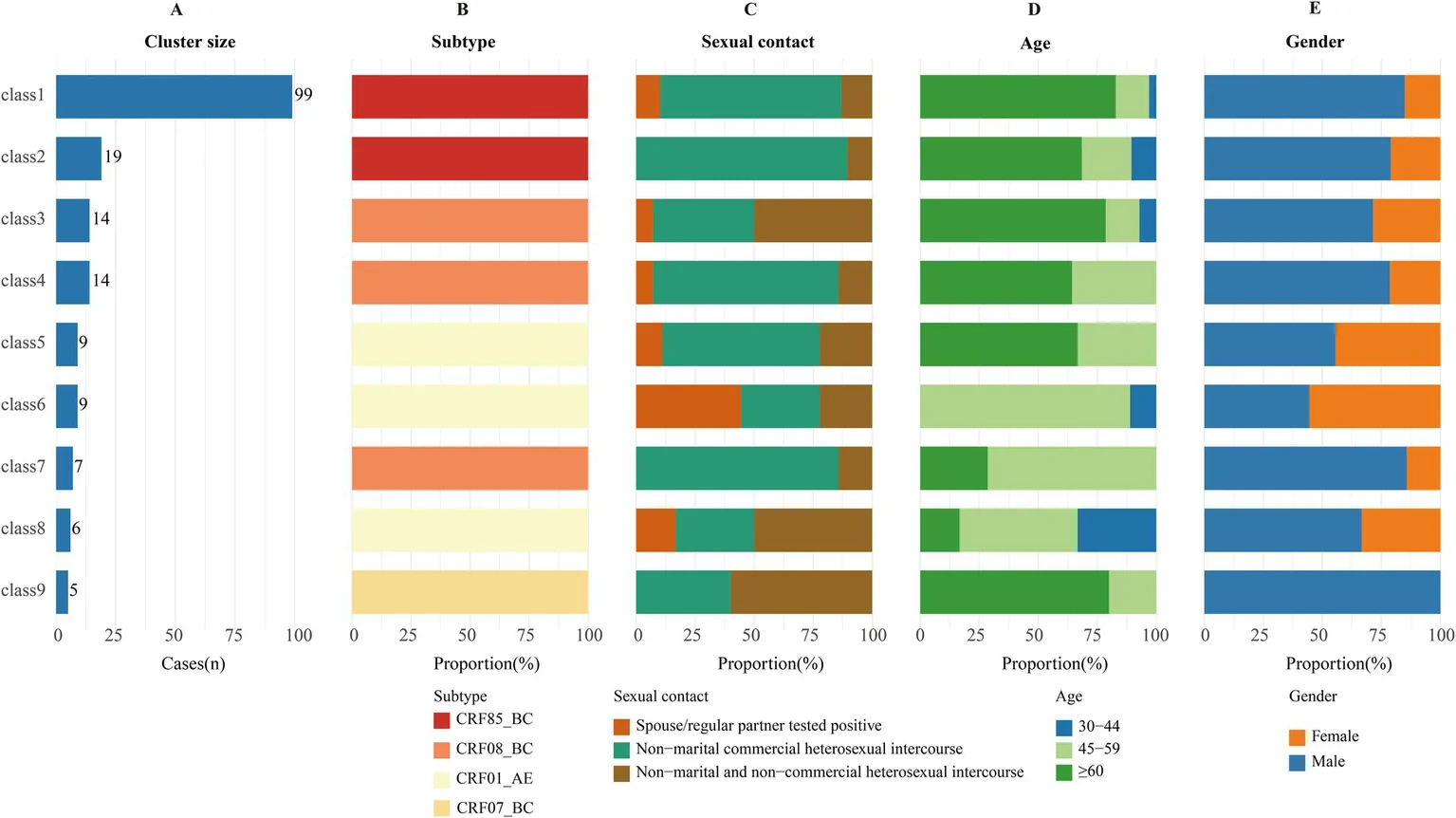
Characteristics of large molecular clusters and medium clusters in the network from 2021 to 2025. **(A)** Sample size of each molecular cluster. Stacked bar charts showing the percentage composition of each cluster by **(B)** HIV-1 subtype, **(C)** sexual contact category, **(D)** age group, and **(E)** gender. Colour codes are provided in each panel legend. Clusters are ordered by decreasing size from top to bottom.

At this genetic distance, a total of 37 transmission clusters were identified. The two largest clusters were found to belong to the CRF85_BC subtype, with 99 and 19 nodes, respectively. The clustering pattern of HIV molecular transmission clusters is demonstrated in Figure 1. Nine transmission clusters, each comprising a minimum of five nodes, were identified. LCs and MCs within molecular networks refer to Table 2 (The remaining clusters are presented in Supplementary Table S1). Cluster 1 accounted for 39.44% (99/251) of all patients in the study who exhibited clustering. The demographic profile of the cases included in the study predominantly comprised males (75.70%, 190/251), individuals aged 60 and above (63.75%, 160/251), farmers (84.46%, 212/251), and non-marital commercial heterosexual transmission (66.53%, 167/251). Figure 2A displays the sample size of each of the LCs and MCs. The stacked bar charts in panels B–E illustrate the proportional distribution of HIV-1 subtypes (B), sexual contact categories (C), age groups (D), and gender (E) within each cluster. CRF85_BC was the dominant subtype in the largest two clusters, and non-marital commercial heterosexual contact was the leading transmission route across all clusters, consistent with the overall demographic profile. Males predominated in every cluster, and individuals aged ≥60 years constituted the largest age group in the majority of clusters.

**Table 2:** Changes in the large molecular clusters and medium clusters in Shaodong, 2021–2025.

Cluster	Year				
	2021	2022	2023	2024	2025
Class1	12	41	65	90	99
Class2	0	10	14	17	19
Class3	1	2	5	13	14
Class4	0	1	5	9	14
Class5	2	5	6	8	9
Class6	4	7	8	8	9
Class7	0	0	0	3	7
Class8	0	3	3	3	6
Class9	0	0	4	4	5

A total of 62 nodes satisfied the high-risk node criteria in this dataset, all with TNS ≥ 0.75 and degrees ranging from a minimum of 17 to a maximum of 64.

Of these, 38 high-risk nodes were male, farmers, and aged ≥60, accounting for 61.29% of all high-risk nodes. This group constitutes the primary high-risk population within the molecular network, necessitating targeted HIV prevention outreach and regular testing for the elderly, particularly older male farmers.

The remaining nodes were comprised of non-agricultural groups, including commercial services, retired personnel, homemakers, and unemployed individuals. Though their numbers are smaller, these groups may serve as conduits for network expansion across populations, necessitating enhanced cross-group tracking.

Eleven female high-risk nodes were identified: the distribution of the sample is as follows: two cases in commercial services, seven cases among farmers, and two cases among homemakers and the unemployed. In certain instances, the context pertains to commercial sex work or household transmission, necessitating the provision of targeted HIV prevention services for women.

### 3.4 Drug resistance characteristics in Shaodong

**Figure 3:**
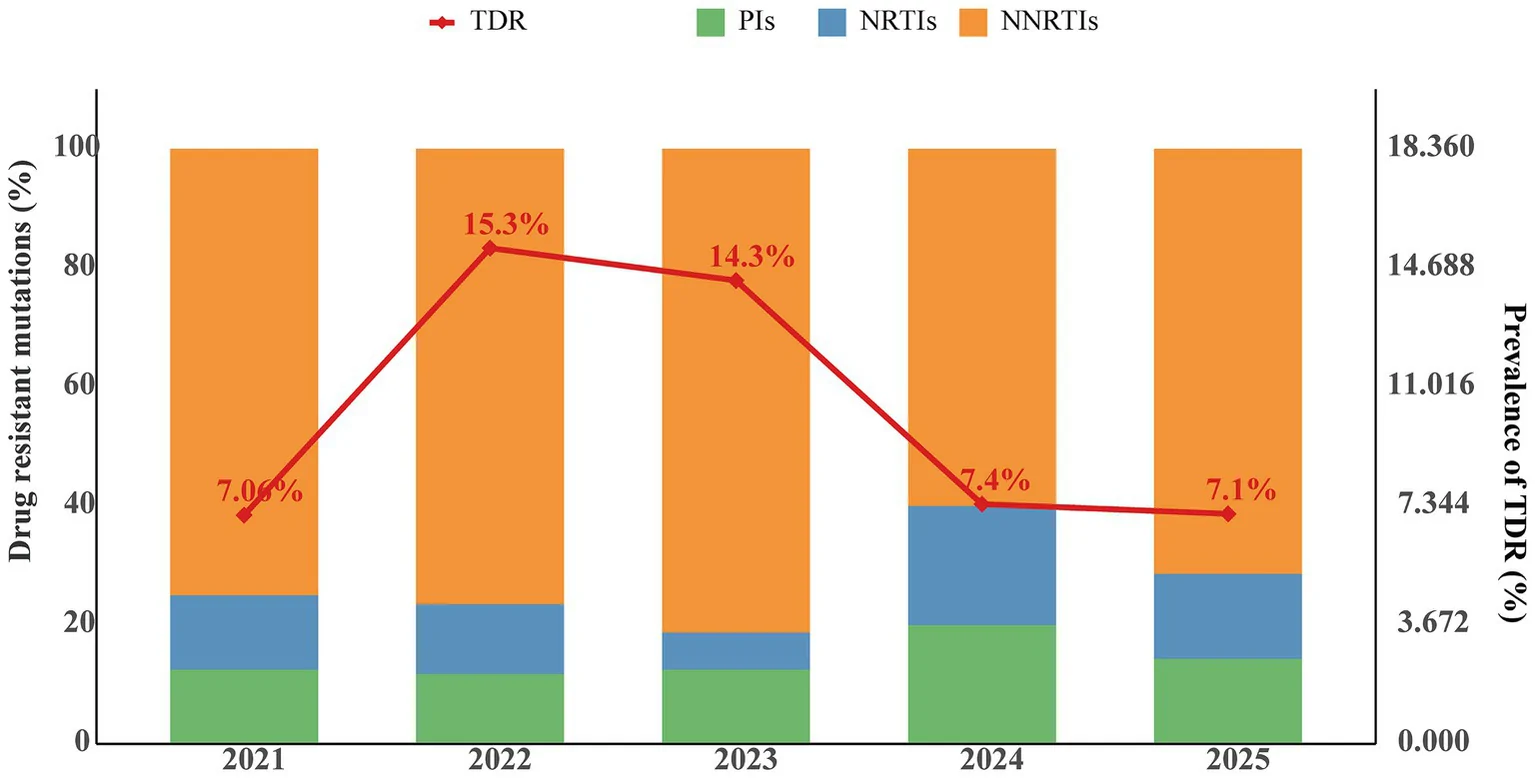
Trends in overall and class-specific TDR prevalence among ART-naive infections, 2021–2025. Stacked bars (left axis) sum to 100%. The overall TDR prevalence (right axis) is plotted on a secondary axis to improve the visibility of its narrow-range fluctuations.

Between 2021 and 2025, the overall prevalence of transmitted drug resistance (TDR) was 10.5% (54/513) in Figure 3. As illustrated in Figure 3, non-nucleoside reverse transcriptase inhibitor (NNRTI) resistance was the most prevalent at 8.4%, followed by protease inhibitor (PI) resistance at 1.6%, and nucleoside reverse transcriptase inhibitor (NRTI) resistance at 1.4%. The prevalence of TDR fluctuated but showed an overall declining trend, peaking at 15.3% in 2022 decreasing to 7.1% by 2023–2025. Single-class resistance accounted for the majority of cases, while dual-class resistance was rare, observed in 2024 and 2025. Triple-class resistance was only observed in 2021.

**Figure 4:**
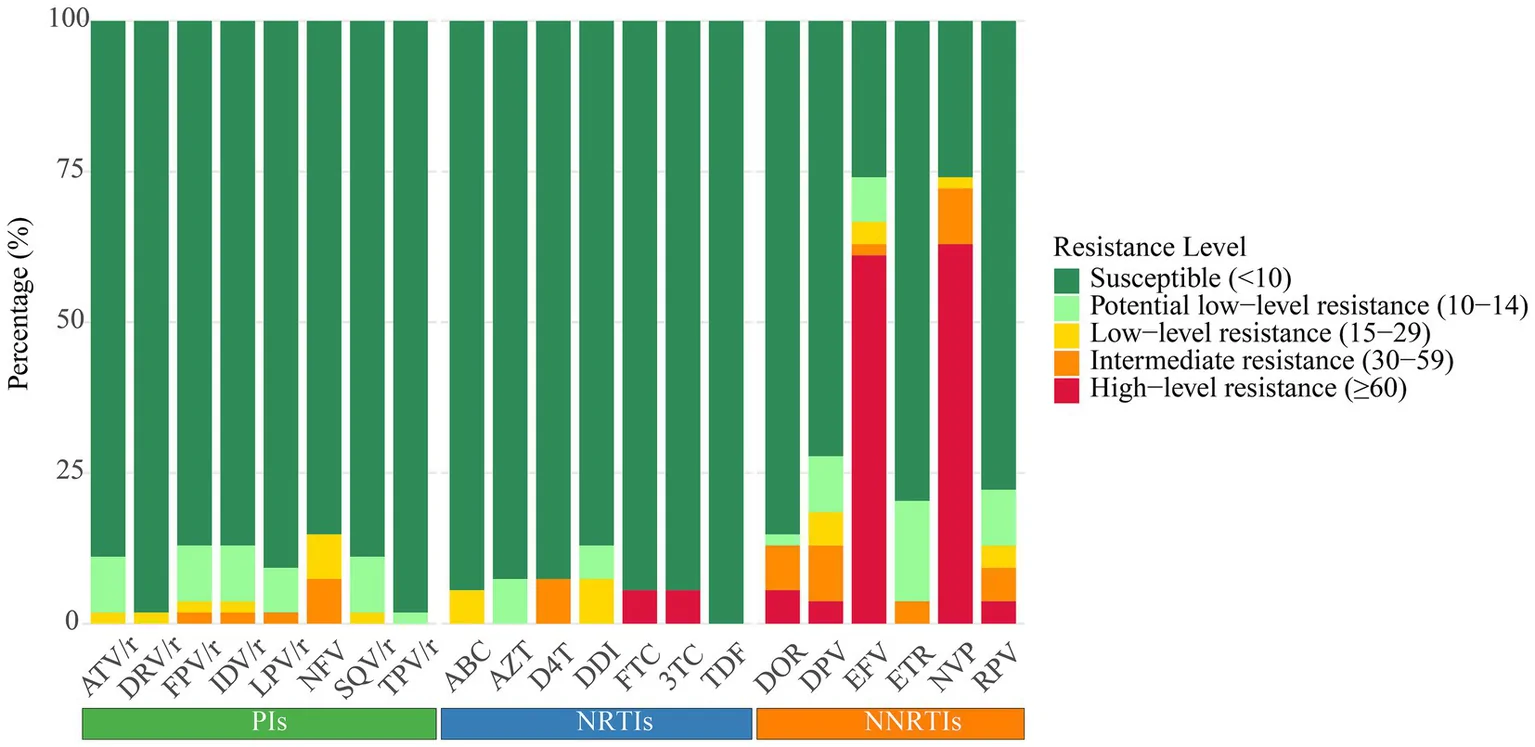
Stacked bar charts showing the percentage distribution of Stanford HIVdb resistance levels for each antiretroviral drug among the 54 cases with transmitted drug resistance (TDR), 2021–2025. Drugs are grouped by drug class (PI, NRTI, and NNRTI). Colour key: Green, susceptible (<10); light green, potential low-level (10–14); yellow, low-level (15–29); orange, intermediate (30–59); red, high-level (≥60).

According to the findings from the HIV Drug Resistance Database(see text footnote 1), the distribution of drug resistance scores for the 54 cases of TDR is shown as stacked bar charts in Figure 4, where the percentage of cases at each resistance level (susceptible to high-level) is displayed for every antiretroviral drug, grouped by drug class (PI, NRTI, NNRTI). We found the highest TDR rates for single-agent drugs were NVP (7.8%, 40/513), EFV (7%, 36/513), DPV (1.9%, 10/513), NFV (1.6%, 8/513), DOR (1.4%, 7/513), RPV (1.4%, 7/513), D4T (0.8%, 4/513), DDI (0.8%, 4/513), ABC (0.6%, 3/513), 3TC (0.6%, 3/513), FTC (0.6%, 3/513), FPV/r (0.6%, 3/513), IDV/r (0.4%, 2/513), ETR (0.4%, 2/513), ATV/r(0.2%, 1/513), DRV/r (0.2%, 1/513), LPV/r (0.2%, 1/513), SQV/r (0.2%, 1/513). Notably, high-level resistance was more prevalent among NNRTIs, with NVP and EFV demonstrating the highest incidence of such resistance. In contrast, resistance to PIs primarily manifested as potential low-level resistance.

**Figure 5:**
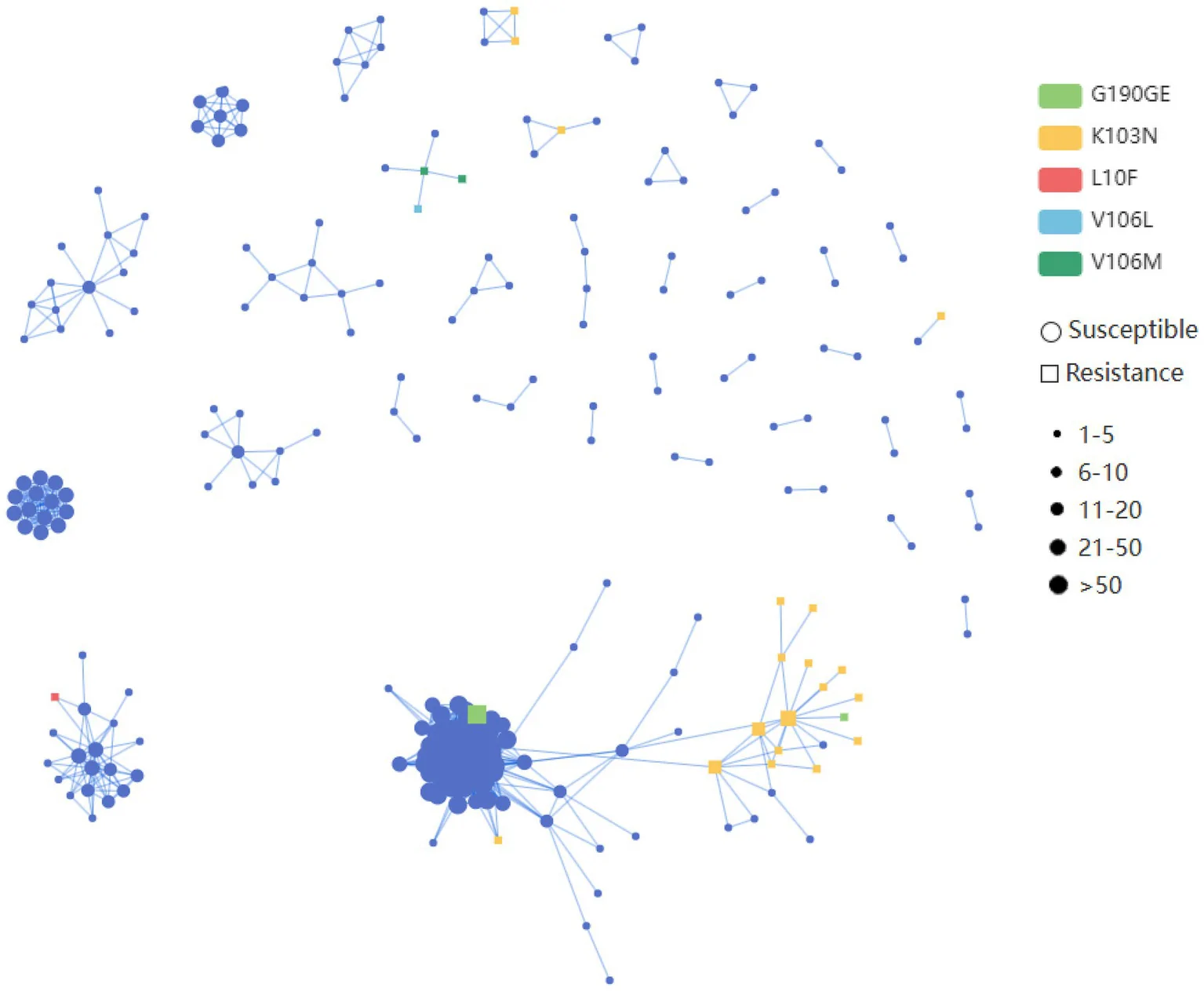
Transmission network by drug resistance mutations in Shaodong.

Among NNRTI resistance mutations, K103N (5.5%, 28/513) had the highest frequency, followed by V106M and E138A (0.6%, 3/513). The most common NRTI resistance mutation was V75M (0.8%, 4/513). For PI resistance, M46MI (0.4%, 2/513) was observed (Supplementary Figure S1). In the transmission network, K103N is the site with the highest mutation frequency (Figure 5).

**Figure 6:**
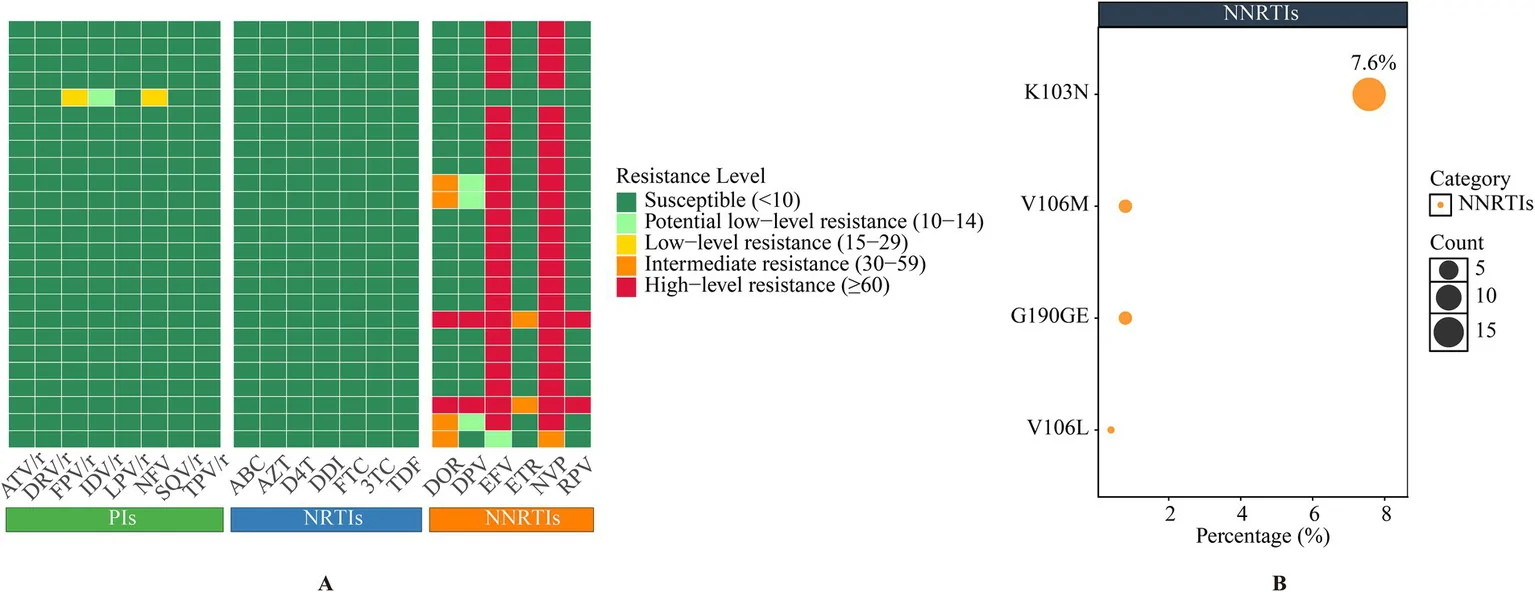
HIV-1 drug resistance within transmission clusters based on the Stanford HIVdb scoring system **(A)** Distribution of drug resistance levels within transmission clusters. In the heatmap, each row represents an individual resistant case from the clusters (*n* = 25), each column represents an antiretroviral drug, and colours indicate the resistance level according to the Stanford HIVdb score **(B)** Prevalence of NNRTI-associated resistance mutations within transmission clusters.

Among the 37 identified transmission clusters exhibiting HIV drug resistance, a total of 25 resistance cases were documented. The drug resistance profiles of these 25 cases are displayed as a heatmap in Figure 6A, where rows correspond to individual cases, columns correspond to drugs, and colours denote resistance levels. Of these, 24 cases (96.0%) demonstrated NNRTI-associated resistance mutations, while 1 case (4.0%) presented with a PI-associated resistance mutation. Notably, the K103N mutation represented the most prevalent NNRTI resistance mutation transmission clusters (Figure 6B).

Beyond describing the overall clustering rate, the molecular transmission network analysis provided additional information on the structure and potential drivers of local HIV transmission. The largest cluster expanded from 12 nodes in 2021 to 99 nodes in 2025, suggesting sustained local transmission rather than isolated introductions. High-risk nodes identified by both TNS and degree were mainly older male farmers aged ≥60 years, indicating that this subgroup may play a central role in maintaining local transmission chains. In addition, several non-agricultural and female high-risk nodes were observed, suggesting potential cross-population transmission and bridge links between commercial sex networks, spouses, and the broader community.

## 4 Discussion

HIV transmission among older adults in Shaodong City is highly clustered, primarily driven by commercial heterosexual contact, with older male farmers constituting the central high-risk nodes.

The inclusion of all age groups enabled a more comprehensive characterization of transmission networks and allowed assessment of potential interactions between older and younger individuals. Nevertheless, the primary emphasis of this study remained the transmission characteristics of older adults, who constituted the majority of clustered cases.

Of the 513 participants, 283 were aged ≥60 years, and 308 reported a history of heterosexual commercial sex. This pattern may reflect older adults’ physiological and psychosocial needs, insufficient dissemination of sexual health knowledge, and misconceptions about HIV-related risk. Collectively, these data indicate suboptimal HIV/AIDS awareness in older populations, together with widespread gaps in knowledge regarding HIV testing and personal susceptibility. Targeted interventions are therefore needed to improve HIV risk perception among older adults, strengthen sexual health education, and promote safer sexual practices alongside accessible, routine testing services.

We found that CRF85_BC was the predominant HIV-1 strain in the Shaodong area, accounting for 32.55% of sequences. This distribution differs from the overall subtype landscape reported for Hunan Province (Zou et al., 2020), suggesting geographic specificity in infection sources, potentially driven by the evolution of sustained local transmission chains and/or distinct introduction pathways. CRF85_BC is a recombinant form generated within China and was first reported in Sichuan Province in 2016. Molecular evolutionary analyses suggest that it likely originated in Yunnan Province, adjacent to Sichuan, and subsequently established local transmission in Sichuan. Since 2020, CRF85_BC infections have been detected in multiple regions across China, including Hunan Province (Zou et al., 2020; Su et al., 2016; Li Y. et al., 2025; Liu et al., 2025b; Yuan D. et al., 2021). At present, CRF85_BC appears largely confined to domestic circulation in China, with few reports elsewhere and no evidence of large-scale international dissemination (Zou et al., 2020; Su et al., 2016; Li Y. et al., 2025; Liu et al., 2025b; Yuan D. et al., 2021). Notably, CRF85_BC is observed almost exclusively in individuals infected via heterosexual transmission and is highly concentrated among older heterosexual adults. Multiple studies have reported that >95% of CRF85_BC infections occur in individuals aged ≥50 years (Liu et al., 2025b; Yuan D. et al., 2021), consistent with our findings. This epidemiological profile is closely linked to HIV acquisition among older men through commercial sexual activity (e.g., paid sex). In our dataset, CRF85_BC formed transmission chains involving older men and their sexual partners (e.g., female sex workers or spouses), highlighting how limited awareness of safer sex and low risk perception among older adults may facilitate sustained transmission.

In addition, we identified several low-prevalence strains, including subtype B, CRF55_01B, CRF97_01B, CRF121_0107, CRF114_0155, and unique recombinant forms (URFs). Prior work suggests that CRF55_01B likely originated around 2003 among men who have sex with men (MSM) in Guangdong Province, and Bayesian skyline analyses indicate an initial increase in effective population size followed by a plateau (An et al., 2025; Zai et al., 2020). CRF97_01B has a CRF01_AE backbone with a subtype B insertion in the gag–pol region and represents the first HIV-1 circulating recombinant form discovered and named in Laos (Chen et al., 2020). Moreover, in 2021, a novel CRF was identified among eight HIV-1–infected individuals in south-central China without direct epidemiological links; recombination analyses showed a five-segment mosaic genome with three segments derived from CRF01_AE cluster 4 and two from CRF55_01B, leading to its designation as CRF114_0155. Bayesian phylogenetic inference dated the most recent common ancestor of the CRF114_0155 clade to approximately 2010, and its emergence underscores increasing complexity in the HIV-1 genotype structure (Li et al., 2021). Taken together, the coexistence of multiple CRFs and URFs reflects substantial genetic complexity and ongoing recombination in this population, implying frequent interactions across subgroups and a challenging transmission environment that requires tailored prevention and control strategies.

Four relatively large clusters were identified in the molecular transmission network, accounting for >58% of clustered cases. This finding indicates that HIV-1 transmission among older adults is not purely sporadic but exhibits pronounced clustering. The clustering rate among older adults infected via commercial heterosexual contact (54.22%, 167/308) was substantially higher than that among individuals infected via MSM (11.11%, 4/36). Previous studies have similarly identified commercial sexual contact as a major risk factor for HIV infection in older adults (Jiang et al., 2021; Chen et al., 2021). Some older individuals may seek commercial sexual services in the context of limited companionship and support from spouses or children, partly to meet psychological and emotional needs (Sun et al., 2022).

In addition, low uptake of proactive testing and a high proportion of late diagnosis have been reported among older adults living with HIV (Justice et al., 2022; Chen et al., 2022), which may prolong the period of unrecognized infection and thereby facilitate onward transmission. In the present study, we did not collect information on the estimated date of HIV infection and therefore could not directly calculate the time from infection to diagnosis. Nevertheless, pre-treatment CD4 cell count at diagnosis was available and was analyzed as an indirect proxy for late diagnosis. Overall, individuals with CD4 < 200 cells/ml were less likely to be incorporated into the molecular transmission network than those with CD4 ≥ 500 cells/ml after multivariable adjustment. This finding suggests that late-diagnosed cases were not overrepresented in the observed molecular clusters and may instead represent longer-standing infections or links to unsampled transmission chains. Therefore, although delayed diagnosis may contribute to sustained transmission at the population level, the relationship between late diagnosis and molecular clustering should be interpreted cautiously in this cross-sectional network analysis.

In our study, nearly half of older adults infected through commercial heterosexual contact were incorporated into the network. We also observed six female sex workers (FSWs) within the network, and their locations were associated with the geographic aggregation of corresponding molecular clusters. These results suggest that commercial sex may play a pivotal role in the transmission network and that older adults may function as a potential “bridge population” facilitating spread from high-risk groups to spouses or the broader community. Targeted interventions should therefore combine routine and accessible HIV testing for older adults, strengthened sexual health education, and focused prevention services for commercial sex networks and their surrounding communities.

We further observed that older age was associated with a higher likelihood of being included in the network. This may be attributable to reduced inter-regional mobility with increasing age, which can promote localized transmission. Moreover, some older adults have lower educational attainment, limited HIV prevention knowledge, weak self-protection awareness, low condom use, and greater engagement in high-risk behaviors, collectively increasing the risk of HIV acquisition and transmission (Sun et al., 2022; Jiang et al., 2018; Yang et al., 2023). Individuals with HIV-positive spouses/regular partners had a higher clustering risk, likely reflecting frequent unprotected sex and insufficient synchronized testing and treatment, which can confine transmission within small networks and facilitate genetic clustering. At the subtype level, CRF85_BC showed a higher clustering risk, plausibly because it is transmitted primarily through sexual contact among older adults—particularly via low-cost commercial sex—combined with low mobility and limited safer-sex awareness, which may facilitate the rapid formation of transmission networks once introduced.

More than 70% of cases within the four large molecular clusters were attributed to heterosexual commercial sex, further underscoring its central role in HIV transmission among older adults. Prior studies have highlighted the presence of low-end commercial sex venues catering predominantly to older men in rural areas, which may contribute to sustained local transmission (Jiang et al., 2021; Deng et al., 2017; Wu et al., 2016). Accordingly, in-depth epidemiological interviews among clustered cases are warranted to identify key individuals and settings that accelerate transmission. Future prevention and treatment strategies—such as increasing condom use and testing frequency, implementing post-exposure prophylaxis (PEP) or pre-exposure prophylaxis (PrEP) in high-risk groups, and rapid initiation of antiretroviral therapy—should adopt a population- and key-network–centered approach.

Although TDR does not influence network construction, the presence of resistant strains within active molecular clusters suggests ongoing transmission of drug-resistant HIV. Therefore, combining molecular network analysis with TDR surveillance may help identify priority clusters requiring enhanced intervention and treatment monitoring. In this study, the overall prevalence of TDR was 10.53%, which falls within the moderate level (5–15%) according to the WHO definition (Bennett et al., 2009). This prevalence was higher than that reported in related studies from Sichuan(5.55%, 72/1297) (Zhou et al., 2022), Guangdong (2.20%, 52/2368) (Lan et al., 2021), and Jiangsu (4.04%, 31/767) (Yin et al., 2021) in China. The prevalence of NNRTI-associated resistance mutations was 8.6%, which was markedly higher than that of NRTI- and PI- associated resistance. This pattern may be attributable to the relatively low genetic barrier to resistance and the propensity for cross-resistance among NNRTIs. Given that NNRTIs constitute a key component of first-line ART regimens in China and are widely used nationwide, the risk of NNRTI-related TDR may be comparatively higher.

We found that NVP and EFV exhibited high-level NNRTI resistance, with K103N being the most frequent mutation. K103N is one of the most common and clinically important resistance mutations among individuals exposed to NNRTIs, and it confers high-level resistance to EFV and NVP, consistent with previous studies (Hong et al., 2023; Zuo et al., 2021). In China, EFV and NVP have been provided as free first-line ART agents since 2004 (Zhou et al., 2011). The long-term and widespread use of these drugs may have facilitated the accumulation of NNRTI-associated resistance mutations among ART-treated individuals, thereby further contributing to the emergence of NNRTI-related TDR. The observed NNRTI-associated TDR likely reflects population-level transmission pressure shaped by historical and ongoing use of NNRTI-based regimens, rather than confirmed transmission from identified ART-experienced partners. As EFV and NVP remain commonly used free drugs in first-line ART regimens in China, the emergence of resistance mutations associated with these agents should be closely and continuously monitored. In particular, baseline resistance testing prior to ART initiation should be implemented to reduce the risk of TDR transmission.

The added value of molecular transmission network analysis lies in its ability to move beyond conventional prevalence-based surveillance. Traditional drug resistance surveillance can identify the frequency of TDR and major resistance mutations, but it cannot determine whether these infections are epidemiologically connected or whether resistant strains are circulating within active transmission clusters. In contrast, the network analysis in this study revealed sustained cluster expansion, identified core high-risk nodes, and demonstrated that NNRTI-associated resistance mutations, particularly K103N, were present within active transmission clusters. These findings provide actionable epidemiological evidence for precision public health interventions, including prioritizing older male farmers, commercial sex-related networks, and clusters containing TDR cases for enhanced testing, prevention, and treatment optimization.

This study has several limitations. First, sequences were not available for all newly diagnosed individuals, and many infected but undiagnosed persons were not captured, which may lead to incomplete epidemiological information and potential bias. Future work should expand sampling coverage to enable more robust subtype-stratified analyses and strengthen inference. Second, we did not evaluate temporal changes in transmission dynamics and spatial patterns; longitudinal studies are needed to better characterize dynamic transmission. Third, our findings primarily reflect the epidemic in Shaodong City. Studies spanning broader geographic regions are warranted, as larger datasets may provide additional insights into HIV transmission among older adults. Fourth, key epidemiological information was self-reported during the initial investigation after HIV diagnosis. Therefore, recall bias and social desirability bias may have introduced some self-reporting bias.

## 5 Conclusion

HIV transmission among older adults in Shaodong City is highly clustered, primarily driven by CRF85_BC and heterosexual commercial sex, with older male farmers as central high-risk nodes. The presence of multiple subtypes and transmitted drug resistance highlights ongoing genetic complexity and potential ART challenges. Molecular transmission network analysis is critical for identifying high-risk individuals and guiding targeted interventions, including prevention, testing, and treatment optimization, to mitigate onward transmission. These findings provide evidence to support precision public health strategies tailored for ageing populations, particularly in regions with elevated commercial sexual activity.

## Data Availability

The datasets presented in this study can be found in online repositories. The names of the repository/repositories and accession number(s) can be found at: https://www.ncbi.nlm.nih.gov/genbank/, PZ460439-PZ460759.

## Footnotes

1
https://hivdb.stanford.edu

2
https://www.hiv.lanl.gov

3
https://nmdc.cn/hiv/
