# Supplementary material for: Molecular transmission network analysis of HIV-1 among older adults in Central China: a cross-sectional study in Shaodong City
Source: Front Microbiol. 2026 Aug 19;17:1876738. doi: 10.3389/fmicb.2026.1876738 (PMC13534127; doi:10.3389/fmicb.2026.1876738)
Supplement: Supplementary file 1 [file Data_Sheet_1.DOCX]

**Fig.S1 Percentage of drug resistance to NNRTIs, NRTIs, and PIs in newly diagnosed HIV-1 infections.**

**Fig.S2 Enlarged view of Cluster 1 (n=99) from the molecular transmission network constructed at a 0.5% genetic distance threshold. (A) Transmission network by subtype and diagnosis year. (B) Transmission network by gender and Sexual contact.**

**Fig.S3. Molecular transmission network constructed using a genetic distance threshold of 1.5% among newly diagnosed HIV-1 infections in Shaodong. (A) Transmission network by subtype and diagnosis year.**
