# Supplementary material for: Molecular transmission network analysis of HIV-1 among older adults in Central China: a cross-sectional study in Shaodong City
Source: Front Microbiol. 2026 Aug 19;17:1876738. doi: 10.3389/fmicb.2026.1876738 (PMC13534127; doi:10.3389/fmicb.2026.1876738)
Supplement: Supplementary file 2 [file Data_Sheet_2.DOCX]

| Table S1 Changes in the remaining clusters molecular transmission cluster nodes in Shaodong, 2021–2025 | | | | | |
| --- | --- | --- | --- | --- | --- |
| Cluster | Year | | | | |
|  | 2021 | 2022 | 2023 | 2024 | 2025 |
| class11 | 2 | 2 | 3 | 4 | 4 |
| class12 | 0 | 2 | 2 | 3 | 4 |
| class13 | 0 | 2 | 2 | 4 | 4 |
| class14 | 0 | 3 | 3 | 3 | 3 |
| class15 | 0 | 0 | 1 | 3 | 3 |
| class16 | 0 | 0 | 0 | 3 | 3 |
| class17 | 0 | 0 | 0 | 3 | 3 |
| class18 | 0 | 0 | 0 | 1 | 3 |
| class19 | 2 | 2 | 2 | 2 | 2 |
| class20 | 2 | 2 | 2 | 2 | 2 |
| class21 | 2 | 2 | 2 | 2 | 2 |
| class22 | 1 | 2 | 2 | 2 | 2 |
| class23 | 2 | 2 | 2 | 2 | 2 |
| class24 | 2 | 2 | 2 | 2 | 2 |
| class25 | 0 | 2 | 2 | 2 | 2 |
| class26 | 0 | 0 | 2 | 2 | 2 |
| class27 | 0 | 0 | 2 | 2 | 2 |
| class28 | 0 | 0 | 2 | 2 | 2 |
| class29 | 0 | 0 | 2 | 2 | 2 |
| class30 | 0 | 1 | 1 | 2 | 2 |
| class31 | 0 | 0 | 0 | 2 | 2 |
| class32 | 0 | 1 | 2 | 2 | 2 |
| class33 | 0 | 0 | 0 | 0 | 2 |
| class34 | 0 | 0 | 0 | 0 | 2 |
| class35 | 0 | 0 | 0 | 0 | 2 |
| class36 | 0 | 0 | 0 | 0 | 2 |
| class37 | 0 | 0 | 0 | 0 | 2 |
